# Waiting for a success story in prevention, control and treatment of Chagas disease in non-endemic countries

**DOI:** 10.1590/0074-02760210277chgsa

**Published:** 2022-04-15

**Authors:** Alessandro Bartoloni

**Affiliations:** 1 University of Florence

Chagas disease (CD) is endemic in the Americas, being present in 21 countries, where it affects about 6 million people.^1^ With such relevant numbers of people affected and disability adjusted life years lost, CD is a poverty-related and poverty-promoting disease.

Although data describe a relevant ongoing public health problem for the American continent, significant results in the interruption of transmission has been achieved by coordinated multi-country programs. In particular, the Southern Cone Initiative (SCI), officially formalised in November 1991 by the Ministers of Health of Argentina, Brazil, Bolivia, Chile, Paraguay and Uruguay, has shown how a well-designed control program can significantly reduce CD transmission.^2^ Before this initiative, in these countries, there were 11 million infected persons and 50 million at risk, 62% of the infected individuals of the whole continent.

Carlos Morel analysed the events from the 1909 historical discovery of Carlos Chagas, to the relevant contribution from other basic and clinical research carried out by a number of scientists, up to the technological and organisational breakthroughs.^3^ Translating this entire scientific heritage into a successful program was possible thanks to a strong political commitment and adequate funds. In this context, Panamerican Health Organization (PAHO) was judged as indispensable for SCI implementation, continuity, and success.^4^

I was impressed when Carlos Pinto Dias, during his excellent speech held at the Consiglio Nazionale delle Ricerche (CNR) in Rome, in June 1991, clearly stated that the needed scientific knowledge was available, as well as the agreement on the appropriate measures to be carried out through an integrated approach to prevent and control CD in endemic areas. Several local and regional efforts to interrupt the transmission had already been successful. The only barrier was the political uncertainty. On 16 May 1998, the World Health Assembly adopted resolution WHA51.4 in which it expressed its satisfaction with the progress made in the countries of the SCI in eliminating the transmission of CD.

The “dream” came true for large regions of the Southern Cone countries, and Morel classifies the story as a paradigmatic case of “success in translation” of the research to political action, inviting to reflect on the lesson learned about the importance of research for improving health.^5^ Nevertheless, a significant reduction in funds for health and sciences and technology, within a scenario of cuts in public funding, was observed in Brazil, and Morel expressed all his regret and concern.^5^

Brazil and other Latin American countries still have to cope with several old and new serious public health threats, with Coronavirus disease 19 (COVID-19) being the current public health emergency. Political commitment and meaningful investments in the provision of health care staff, resources, and systems, as well as in training and capacity building for research and development, are expected to prevent, control, and mitigate neglected tropical diseases and other public health threats and to help create sustainable models for the future.

From the perspective of health care providers, researchers and policy makers of non-endemic countries (NEC), CD has been considered for many years as a health problem limited to Latin America. In NEC, educational programs for health professions have dedicated, for a long time, a limited, if any, space to CD. The majority of teachers were not familiar with this disease. We could realise that when some of them mentioned CD with the French pronunciation *Chagà*, as if Carlos Chagas was a French scientist.

In Europe, the establishment of tropical medicine as a scientific and academic discipline is strictly related to colonial expansionism in the tropical areas of Africa and Asia.

Even within the tropical medicine expert community, CD has received little attention for a long time. The story is different for the Human African trypanosomiasis (HAT), also known as “sleeping sickness”, caused by the species *Trypanosoma brucei* and transmitted by infected tse-tse flies, that attracted considerable scientific research and political attention. HAT represented a major obstacle to the economic development of the European colonies in Sub-Saharan Africa.^6^

Lack of awareness and information on CD is now a problem for health care systems in Europe and other NEC, such as North America (United States and Canada) and the western Pacific region (mainly Japan and Australia), considering the sustained migration of Latin American people, particularly during last decades.

CD represents an important public health challenge due to the presence of a remarkable number of chronically infected people who will need medical treatment for decades to come, and to the possibility to observe vertical, transplant- and transfusion- related cases.^7^ The increasing trend towards the feminisation of migration is also relevant for congenital transmission of *T. cruzi* infection.^8^

The response to the worldwide spread of CD, known as a neglected disease of poor, rural, and forgotten people of Latina America, implies a series of interventions starting from breaking of the epidemiological silence, intended as lack of detected cases.^9^ Underdiagnosis is in part related to the little or no experience with the detection and management of CD of most European health professionals, but also to the lack of an efficient approach to case finding and transmission control in many countries.

It is evident that priority actions are the implementation of screening programs of target populations and of national and international surveillance systems, as well as the training of professionals in the detection of possible cases and in their appropriate management. Reliable data on documented and undocumented migrants from endemic countries should be available. Barriers to the access of health care services should be identified in order to develop and implement practical solutions for healthcare professionals, health authorities and governments to help overcome them. Any effort should be made to include undocumented immigrants, at risk of being excluded from the national health systems. Psychosocial barriers, such as fear of the disease, and stigma must also be taken into consideration.

Community interventions have been reported as successful when implemented in Barcelona (Catalonia, Spain).^10^ Cooperation with and involvement of patients’ associations and community is useful to access to individuals at risk, providing information and improving awareness of CD. In this perspective, the International Federation of Associations of People Affected by Chagas Disease (FINDECHAGAS), was founded in 2010, bringing together more than 20 associations of affected people in the Americas, Europe, and the West Pacific.

Recently, screening asymptomatic adult Latin American migrants for CD in Europe and treating *T. cruzi*-seropositive individuals with antiparasitic therapy has been found a cost-effective strategy.^11^

In the setting of travel medicine, raising awareness on oral transmission of CD will allow staff to correctly inform travelers to endemic areas on the risk and prevention measures, and to recognise the early symptoms of acute CD in patients returning from Latin American countries.

It is evident that CD must be taken seriously with a global perspective and a success story in the prevention and control of CD is now waited from NEC.

In 2007, the World Health Organization (WHO) and PAHO convened a meeting of endemic Latin American countries and non-Latin American countries. A major outcome of the meeting was to highlight the presence of *T. cruzi* infection outside Latin America. Recognising the globalisation of CD, the 28 participating countries called for the establishment of an additional initiative to deal with CD in the NEC.

WHO held an informal consultation on the control and prevention of CD in Europe at its headquarters in Geneva, Switzerland, on December 2009. The meeting was jointly organised by WHO headquarters and the WHO Regional Office for Europe. A total of 31 participants representing nine countries, WHO and the Special Program for Research and Training in Tropical Diseases attended the meeting. The aims of the meeting were to build consensus on the implementation of national measures to prevent and control CD in Europe and to target the harmonisation of national policies at the European level through recommendations to European and national authorities.^8^

Different activities are currently ongoing in NEC to better define the magnitude of CD burden, to implement national surveillance systems, to promote screening programs to target populations, to map the national diagnostic capacity, to evaluate strategies to improve the access to diagnosis and treatment of CD, to raise awareness among health care personnel, public and policy makers.^8^ Research activities are expected to provide further important results to fill knowledge gaps and to help define the appropriate strategies to prevent, control, diagnose and treat CD, including cost-benefit evaluation.

Diagnostic procedures, as well as procedures for treatment and clinical management, should be harmonised, validated and spread through appropriate guidelines.^8^ The same harmonisation process should be followed in the implementation of national policies.

NEC have the opportunity to write another successful story on CD, addressing this new public health threat generated by the migrant flux from Latin America.

Following the SCI experience, a scientific consensus on the strategies to be used would be desirable and sharing protocols and legislation, when successfully implemented in some countries, could reach this.

As PAHO had a key role in the SCI implementation, continuity, and success, WHO is now supporting healthcare professionals, health authorities and governments towards the implementation of the necessary measures to stop CD transmission and provide care to patients in Europe, as well as in the other NEC.

The worldwide spread of CD is certainly a critical challenge that must be addressed seriously but, at the same, it may represent an opportunity to generate or reinforce international network activities of academic and research institutions, within a perspective of south-north and north-south collaboration. The ultimate beneficiaries of a worldwide multidisciplinary effort will be the affected neglected populations in both endemic and non-endemic countries.
